# Prevalence and determinants of anti-tuberculosis treatment non-adherence in Ethiopia: A systematic review and meta-analysis

**DOI:** 10.1371/journal.pone.0210422

**Published:** 2019-01-10

**Authors:** Abriham Zegeye, Getnet Dessie, Fasil Wagnew, Alemu Gebrie, Sheikh Mohammed Shariful Islam, Bekele Tesfaye, Dessalegn Kiross

**Affiliations:** 1 Department of Biomedical Sciences, School of Medicine, Debre Markos University, Debre Markos, Ethiopia; 2 Lecturer of Nursing, Department of Nursing, College of Medicine and Health Sciences, Bahir Dar University, Bahir Dar, Ethiopia; 3 Department of Nursing, College of Health Sciences, Debre Markos University, Debre Markos, Ethiopia; 4 Institute for Physical Activity and Nutrition (IPAN), Deakin University, Geeland, Australia; 5 Sydney Medical School, University of Sydney, Sydney, Australia; 6 Department of Psychiatry, College of Medicine and Health Science, Adigrat University, Ethiopia; Baylor College of Medicine, UNITED STATES

## Abstract

**Background:**

Tuberculosis is a global public health problem. One of the overarching dilemmas and challenges facing most tuberculosis program is non-adherence to treatment. However, in Ethiopia there are few studies with variable and inconsistent findings regarding non-adherence to treatment for tuberculosis.

**Methods:**

This systematic review and meta-analysis was conducted to determine the prevalence of non-adherence to tuberculosis treatment and its determinants in Ethiopia. Biomedical databases including PubMed, Google Scholar, Science Direct, HINARI, EMBASE and Cochrane Library were systematically and comprehensively searched. To estimate the pooled prevalence, studies reporting the prevalence of adherence or non-adherence to tuberculosis treatment and its determinants were included. Data were extracted using a standardized data extraction tool prepared in Microsoft Excel and transferred to STATA/se version-14 statistical software for further analyses. To assess heterogeneity, the Cochrane Q test statistics and *I*^*2*^ test were performed. Since the included studies exhibited high heterogeneity, a random effects model meta- analysis was used to estimate the pooled prevalence of non-adherence to tuberculosis treatment. Finally, the association between determinant factors and non-adherence to tuberculosis treatment was assessed.

**Results:**

The result of 13 studies revealed that the pooled prevalence of non-adherence to tuberculosis treatment in Ethiopia was found to be 21.29% (95% CI: 15.75, 26.68). In the subgroup analysis, the highest prevalence was observed in Southern Nations and Nationalities of Ethiopia, 23.61% (95% CI: 21.05, 26.17) whereas the lowest prevalence was observed in Amhara region, 10.0% (95% CI: 6.48, 13.17.0;). Forgetfulness (OR = 3.22, 95% CI = 2.28, 4.53), fear side effect of the drugs (OR = 1.93, 95% CI = 1.37, 2.74), waiting time ≥ 1 hour during service (OR = 4.88, 95% CI = 3.44, 6.91) and feeling distance to health institution is long **(**OR = 5.35, 95% CI = 4.00, 7.16) were found to be determinants of non-adherence to tuberculosis treatment.

**Conclusion:**

In this meta-analysis, the pooled prevalence of non-adherence to tuberculosis treatment in Ethiopia was high. Forgetfulness, fear of side effect of the drugs, long waiting time (≥1 hour) during service and feeling distance to health institution is long were the main risk factors for non-adherence to tuberculosis treatment in Ethiopia. Early monitoring of the side effects and other reasons which account for missing medication may increase medication adherence in patients with tuberculosis in Ethiopia.

## Background

Tuberculosis (TB) is a communicable disease caused by the bacillus *Mycobacterium tuberculosis*. It usually affects the lungs (pulmonary TB) and transmitted when people who are sick with pulmonary TB expel bacteria into the air, but can also affect other sites (extra pulmonary TB) [1].

TB is a global public health problem; approximately one third of the world population is infected by Tubercle bacilli and at risk of developing active disease [2]. In 2016, globally there were an estimated 10·4 million new cases of tuberculosis, and 600 000 new cases with resistance to Rifampicin (the most powerful first-line drug) [3]. Ninety percent of total TB cases and deaths occur in the developing world. Among those, 75% of cases are in the most economically productive age group [4].

According to the World Health Organization (WHO) TB control report, among the world’s 22 high-burden TB countries, Ethiopia ranks seventh [5]. Agreeing to the ministry of health, TB is the second cause of death after malaria, the third cause of hospital admissions and a main reason of morbidity in Ethiopia. The expected TB incidence in Ethiopia was 261/100,000 inhabitants in 2011. In Ethiopia, almost 50–60% of people living with HIV and 10% HIV-negative people have life-time risk of developing TB [6, 7].

Worldwide, the implementation of Directly Observed Treatment (DOT) has been related to decreased rate of treatment failure, relapse and drug resistance. However, its influence on decreasing TB incidence has been inadequate by non-compliance to DOT, which happens when patients do not turn up for treatment at health facility or community DOT points [8]. According to the WHO’s report on worldwide plan to halt TB, poor treatment has been caused by evolution of *Mycobacterium tuberculosis* strains that do not respond to treatment with standard first line combination of anti-TB medicines, resulting in the emergence of multi drug resistance TB (MDR-TB) in almost every country of the world [5]. One of the overarching dilemmas and challenges facing most TB program’s is a patient that does not complete TB treatment for one reason or another [9].

There is an unfavorable magnitude of poor adherence to treatment of chronic diseases including TB in the world [10]. However, greater than 90% of patients with TB are expected to adhere the treatment in order to facilitate cure. Poor adherence to treatment results failure of cure which increases the risk of development of drug resistant strains, spread of TB in the community and this in turn increases morbidity and mortality [11, 12].

Although many national and international efforts have been implemented against TB prevention and control, still patients are failing to complete their treatment to declare cure even with execution of globally recommended strategy (DOT) in almost all parts of the WHO regions [13–15]. According to the current WHO report, throughout the world, significant TB patients have not recovered after several treatments. Some of obstacles are defaulting from treatment, relapsing after completion of the treatment and developing MDR-TB among retreatment cases [16].

In developing countries, particularly, there are many factors affecting adherence to TB treatment as evidenced from a variety of literatures. Age, lack of treatment support from family, extreme illness, far distance, lack of access to formal health services, traditional beliefs leading to self-treatment, low income, lack of social support, drug side effects, pill burden, lack of food, stigma with lack of disclosure, and lack of adequate communication with health professionals were some of the documented factors [5, 17–21].

In Ethiopia, different studies have been conducted to determine the prevalence and determinants of non-adherence to TB medications [22–34]. Those studies reported that the prevalence of non-adherence among TB patients ranging from 8.4% to 55.8%. This is higher than the WHO recommendation of less than 10% [35]. Variable and inconsistent prevalence and determinants of non-adherence were observed in the reported studies. Therefore, this systematic review and meta-analysis aimed to estimate the pooled prevalence of non-adherence to TB treatment and its determinants in Ethiopia. A better understanding of the magnitude and reasons for non-adherence to TB treatment will help in planning and implementing future strategies for the control of the disease.

## Methods

### Data sources and search strategy

Studies reporting the prevalence of non-adherence to TB treatment in Ethiopia were systematically and comprehensively searched using biomedical databases; PubMed, Google Scholar, Science Direct, HINARI, EMBASE and Cochrane Library according to Preferred Reporting Items for Systematic Reviews and Meta-analysis (PRISMA) guideline [36].

All published and unpublished original articles with epidemiological data on the prevalence and associated factors of adherence or non-adherence to TB treatment conducted in Ethiopia from 1980 to February 2018 were included in this review. Manual searches for additional relevant studies using references from retrieved articles were also performed. Studies were searched by using the following keywords separately or in combination: “Tuberculosis”, “Adherence to TB medication”, “Non-adherence to TB medication”, “Ethiopia”, “Prevalence” and “Associated factors”. The search terms were used separately and in combination using Boolean operators like “OR” or “AND” **(S1 File).**

### Inclusion criteria

Studies which fulfilled the following criteria were included after systematically reviewing the content of the manuscripts.

Publication type: journal articles, master’s thesis and dissertations
Setting: community or institutional based studies in different regions of Ethiopia
Participants: people of all ages, regardless of their sex and occupation.
Language: articles published in English language.
Study design: studies that assessed the prevalence and associated factors of non-adherence to TB medications.
Study area and period: only studies conducted in Ethiopia from 1980 to February 2018 were included in the review.

### Exclusion criteria

- Conference abstracts
- Articles which assessed non-adherence to TB treatment among people living with HIV only
- Articles with incomplete information, methodological problems or no full text available were also excluded, because this may limit the analyses.

### Data extraction and quality assessment

The data extraction was done by two investigators independently using Microsoft Excel with a tool. This tool included information on the author, year of publication, region, study design, sample size, included participants, and number of patients with non-adherence treatment, TB prevalence rates and risk factors significantly related to non-adherence in TB patients.

Among the articles identified, titles and abstracts were reviewed to retrieve studies on the prevalence of non-adherence to TB treatment. Articles found relevant by title and abstract screened for full text review for eligibility. The quality of eligible studies was assessed against predefined inclusion criteria and the quality assessment tool for cross sectional studies known as Newcastle-Ottawa Scale [37]. High quality articles were determined if scale score was 6 and above out of 10. Two investigators conducted the selection, extraction and quality assessment of articles independently. Inconsistencies between the investigators were solved by discussion and articles were included after consensus.

### Outcome measurement

Anti-TB treatment adherence status was the outcome variable dichotomized as adherent and non-adherent. A patient belonging to either intensive or continuation phase under new or retreatment regimen who missed ≥10% of the total prescribed dose was considered as non-adherent. For sputum follow up, patients who missed one and more sputum tests were considered as non-adherent. The primary outcome of this study was the prevalence of non-adherence to TB treatment. The prevalence was calculated by dividing the number of TB patients who did not adhere by the total number of study subjects multiplied by 100. The second outcome considered in this systematic review and meta-analysis was a risk factor that is associated with non-adherence to TB treatment. For the analysis of the risk factors, we considered and collected risk factors which were mentioned in two or more studies by looking at the two by two tables of the factors reported in each study. E-mails were sent to the corresponding or first authors of the studies or abstracts for missing information and a waiting time of 3–4 weeks were taken for the responses. If there were no responses, we excluded the study or the parameter that was not available.

### Statistical methods, publication bias, heterogeneity and analysis

The necessary information from each original study was extracted by using a format prepared in Microsoft Excel spreadsheet. Then, the data were transferred into STATA/se version-14 statistical software for further analyses. For studies which did not present it, a standard error (SE), was calculated using the formula: SE = √p x (1-p)/n in Microsoft excel. The calculated standard error and prevalence rate of each study was then entered into STATA/se version-14 software to calculate the overall prevalence and its 95% CI.

Publication bias and heterogeneity were also assessed. To check publication bias, a funnel plot, Egger’s and Begg’s tests at 5% significant level were performed. The distribution of studies and a p-value <0.05 were used to declare publication bias. The heterogeneity of studies was checked using Cochran’s Q test (P < 0.10 indicated statistically significant heterogeneity) and I^2^ test statistics (which shows the amount of heterogeneity between studies). A naive categorization of values for *I*^2^ would not be appropriate for all circumstances, although we would tentatively assign adjectives of low, moderate, and high to *I*^2^ values of 25%, 50%, and 75% respectively [37]. The test result indicated the presence of significant heterogeneity; as a result, a random effects model was used to estimate the Der Simonian and Laird's pooled effect.

The pooled effect size was computed in the form of prevalence and odds ratio. To minimize the random variations between the point estimates of the primary study, subgroup analysis was carried out based on the geographical settings (regions). In addition, to identify the possible source of heterogeneity, univariate meta regression was performed by considering the sample size of each study. Furthermore, point prevalence with 95% CIs were presented in forest plot. In this plot, the size of each box indicates the weight of the study, while each crossed line refers to 95% CI. To identify possible determinants, pooled effect was articulated in the form of odds ratio. Sensitivity analysis was also performed to identify the primary determinant of the pooled result and the main source of heterogeneity.

## Results

### Literature searches and selection

**Fig 1:**
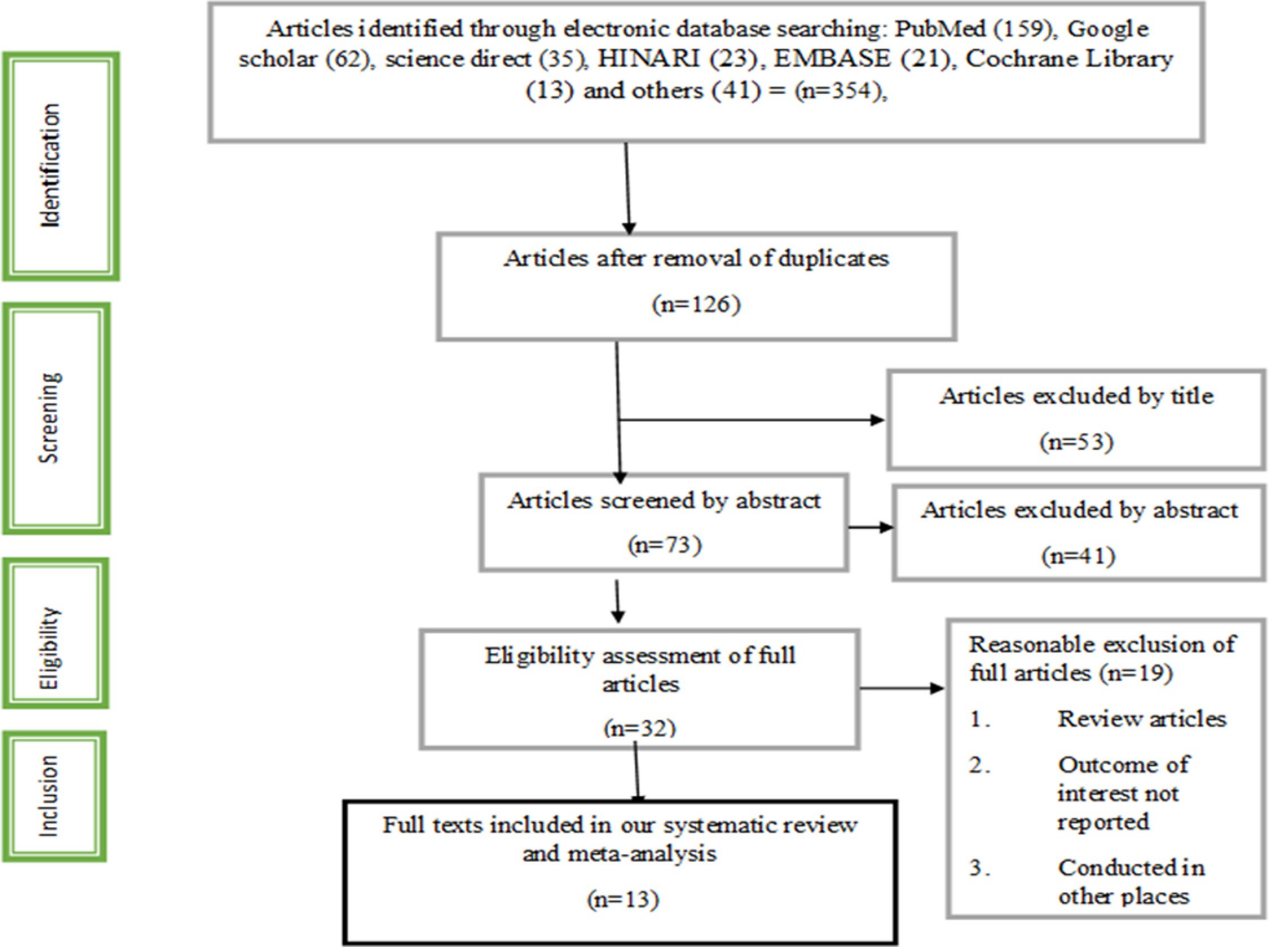
Flow chart diagram describing selection of studies for the systematic review and meta-analysis of prevalence and determinants of non-adherence to anti TB medication in Ethiopia, 2018.

We identified 354 articles in the electronic search databases of MEDLINE/PubMed, Google Scholar, Science Direct, HINARI, EMBASE, Cochrane Library and reference lists of previous relevant studies to identify more related literatures in the initial screening. From these, 126 records remained after removing duplication. After assessing and screening the titles and abstracts, 53 records were excluded. We assessed the full texts of 32 remaining records for eligibility, and 19 records were further excluded for not fulfilling the inclusion criteria. Finally, 13 of the retrieved studies were included in the meta-analyses [22–34] (Fig 1).

### Characteristics of the original articles

Table 1 summarizes the characteristics of 13 original articles included in this systematic review and meta-analysis. Twelve of 13 articles were cross sectional studies and one was prospective cohort study design conducted in Ethiopian TB patients [38]. Sample size ranged from 24 in Tercha District Hospital in South Ethiopia [26] to 404 in Hosanna Zonal Hospital in Hadiya zone of the southern region of Ethiopia [38]. All studies were carried out between 2007 and 2017. A total of 2797 Ethiopian TB patients were included to estimate the pooled prevalence of non-adherence to anti TB medication in the present meta-analysis. The studies were conducted in different regions of the country: Amhara [28], Tigray [22, 29, 30, 32], Oromia [24, 33, 34] and Southern Nations and Nationalities of Ethiopia [23, 25–27, 31].

**Table 1:** Summary of included studies evaluating the prevalence and risk factors of non-adherence to anti TB medication in Ethiopia.

Author, Year	Study design	Region	Sample size	Response rate (%)	Prevalence (95% CI)	Quality score
Nezenga ZS et al, 2013	Cross-sectional	SNNP	531	95.5	26.00 (22.18, 29.82)	5
Woimo TT et al, 2017	Cross-sectional	SNNP	271	96.3	24.50 (19.28, 29.72)	7
Adane AA et al, 2013	Cross-sectional	Amhara	280	99.6	10.00 (6.48, 13.52)	6
Kebede A et al, 2012	Cross-sectional	SNNP	24	100	20.80 (4.56, 37.04	7
Getachew *et al*, 2015	Cross-sectional	Tigray	162	95	18.00 (11.93, 24.07	5
Yusuf KO et al, 2015	Cross-sectional	Oromia	126	72.2	29.67 (20.28, 39.06)	7
Daksa et al, 2016	Cross-sectional	Oromia	67	100	12.00 (4.22, 19.78)	7
Tesfahuneygn *et al*, 2015	Cross-sectional	Tigray	200	100	11.50 (7.08, 15.92)	7
Gubie AA et al, 2017	Cross-sectional	SNNP	281	96.4	24.70 (19.57, 29.83	5
Shargie EB et al, 2007	Cohort	SNNP	404	97.8	20.00 (16.04, 23.96)	6
Eticha T et al, 2014	Cross-sectional	Tigray	120	100	55.80 (46.91, 64.69)	7
Ahmed Yasin et al, 2014	Cross-sectional	Oromia	53	100	20.70 (9.79, 31.61)	7
Kiros YK et al, 2014	Cross-sectional	Tigray	278	98	8.40 (5.10, 11.70	5

Abbreviations: SNNP Southern Nations Nationalities and Peoples, TB Tuberculosis, CI Confidence Interval

### Meta-analysis

**Fig 2:**
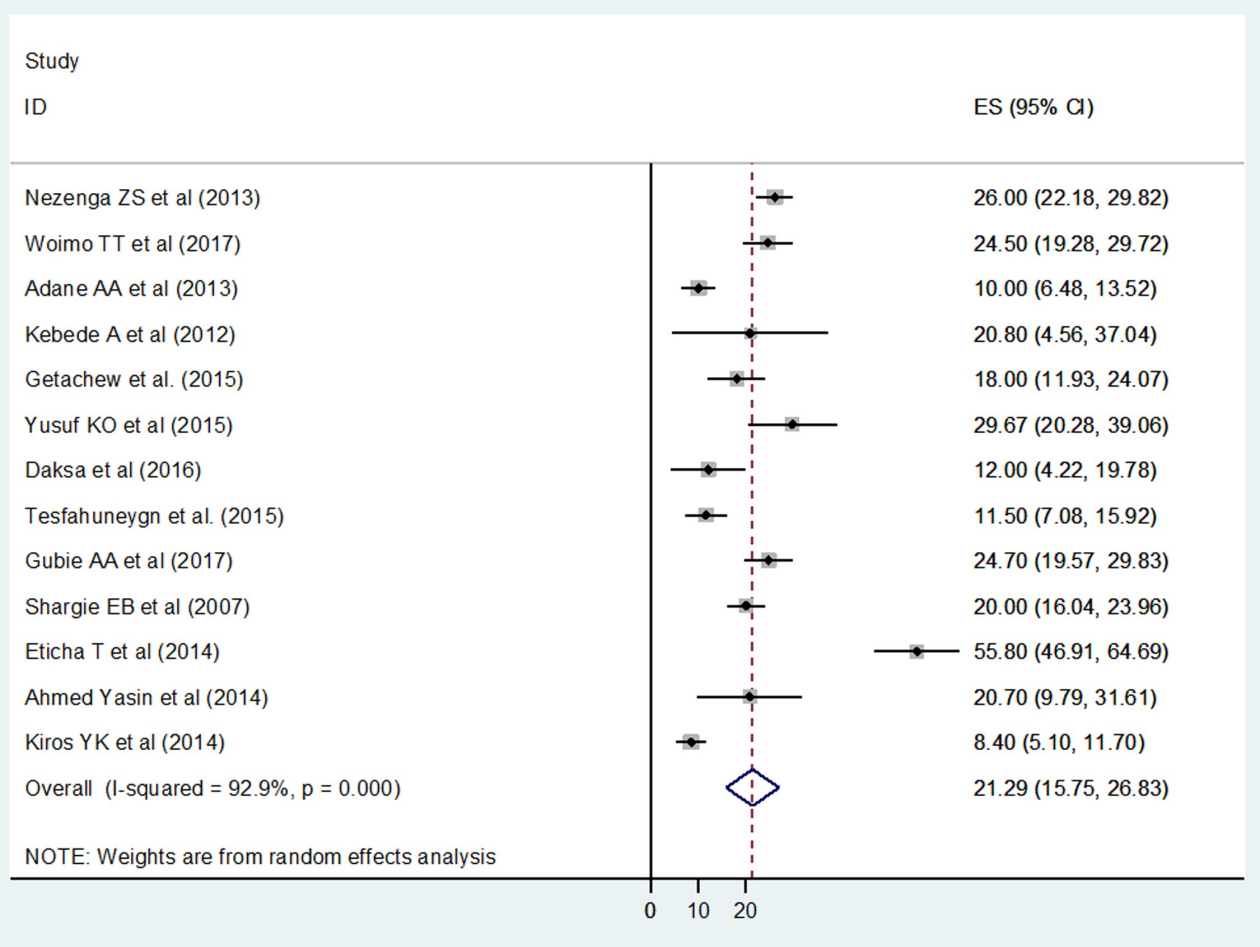
Forest plot depicting the pooled prevalence of non-adherence to anti TB medication in Ethiopia.

Thirteen studies were included for the analysis to determine the prevalence of non-adherence to anti TB medication. The overall pooled prevalence was found to be 21.29% (15.75, 26.83) (Fig 2). The test statistic showed a high heterogeneity among the studies (I^2^ = 92.9%, p<0.001). As a result, random effects model was used to estimate the Der Simonian and Laird's pooled effect.

**Fig 3:**
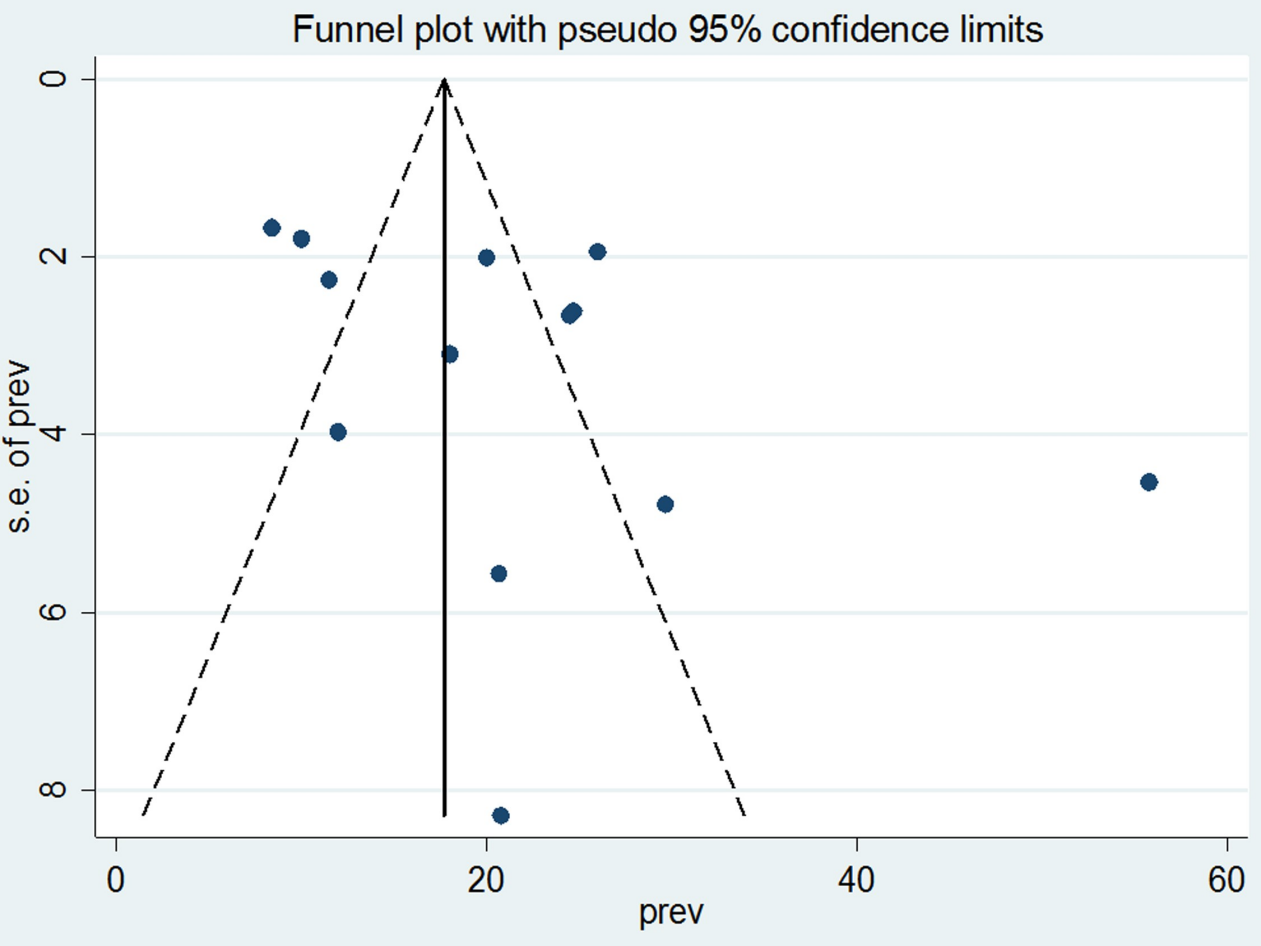
Funnel plot to assess publication bias for non-adherence to anti TB treatment in Ethiopia.

We assessed publication bias by looking at the funnel plot for symmetry by visual inspection (Fig 3). The funnel plot appeared quite symmetrical and showed no publication bias. The Egger weighted regression (p = 0.103) and Begg rank correlation test (p = 0.428) also revealed evidence of no publication bias.

**Fig 4:**
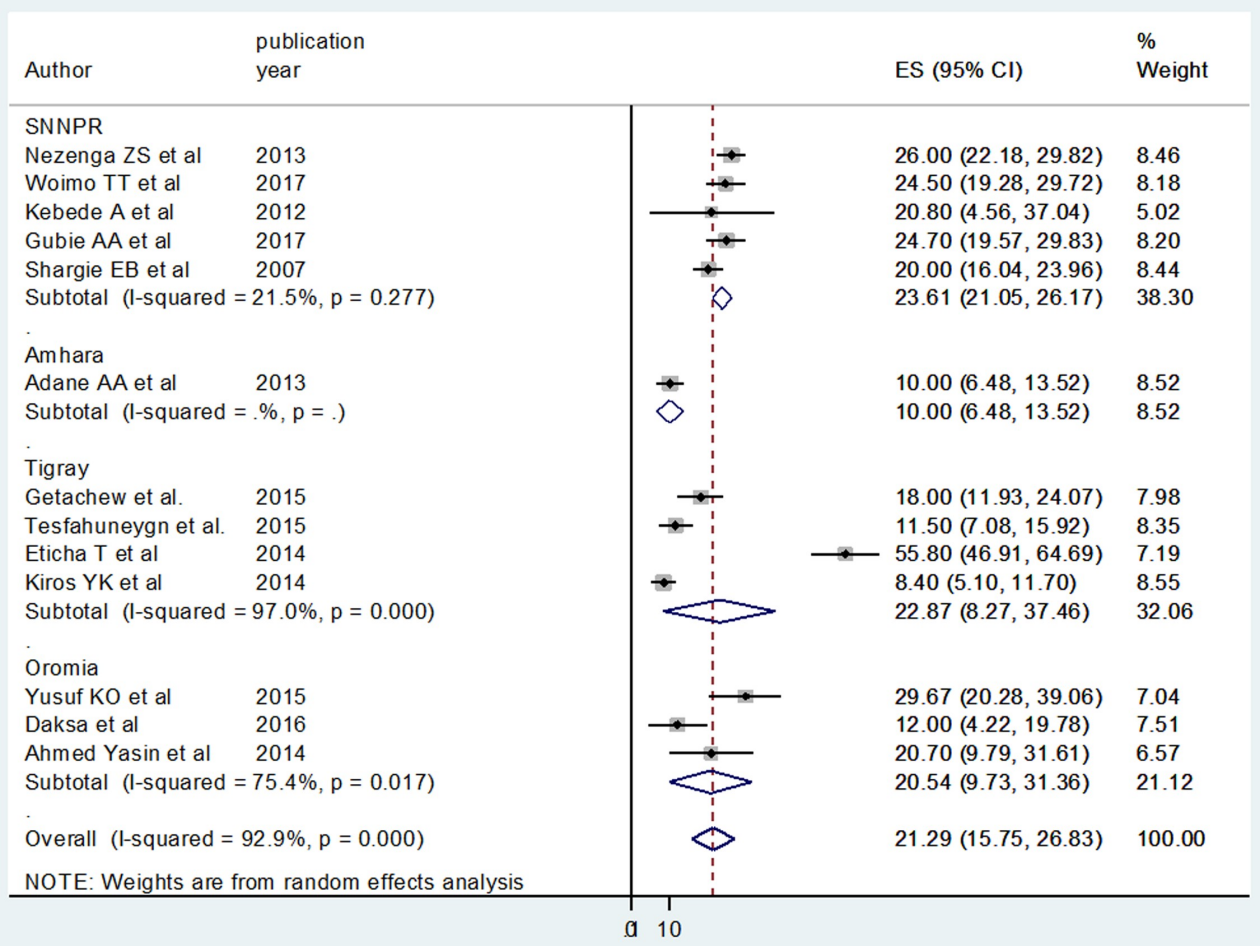
Forest plot depicting subgroup analysis of non-adherence to anti TB medication in Ethiopia according to the location of the studies assessed.

In addition, in this meta-analysis, we preformed subgroup analyses based on the geographical setting (region of the country) of the studies. The pooled prevalence estimate of non-adherence to anti TB treatment was highest in the Southern Nations and Nationalities of Ethiopia, 23.61% (95% CI: 21.05, 26.17), followed by Tigray region with a prevalence of 22.87% (95%CI: 8.27, 37.46) and the least was in Amhara region, 10.0% (95% CI: 6.48, 13.17.0) (Fig 4).

### Risk factor analysis

The risk/associated factors for tuberculosis non-adherence were heterogeneous. Eight of studies included a discussion of risk factors associated with non-adherence. The remaining five studies did not incorporate risk factor analysis. These risk factors frequently discussed in the majority of the studies were employment status, educational status, relationship with health workers, proper counseling, means of transportation, forgetfulness, fear of side effect of the drug, long waiting time in health institutions and feeling distance to health institution is long. A number of studies concluded that non-formal education increases the risk of non-adherence to TB treatment [25, 27, 33, 34]. Poor knowledge and poor counseling services were also reported as factors associated with non-adherence to TB treatment [22, 25, 33]. On the contrary, good relationship with care providers was reported as a low risk factor for non-adherence to TB [23, 25, 33, 34]. Being farmer and transportation on foot to health institutions were reported as increased risk factors for non-adherence to anti TB treatment [23, 25, 30]. We were able to perform a meta-analysis on four of the risk factors. The pooled odds ratios ranged from 1.93 (fear of side effect of the drug) to 5.35 (distance to health institution).

### Forgetfulness and non-adherence of anti TB treatment

**Fig 5:**
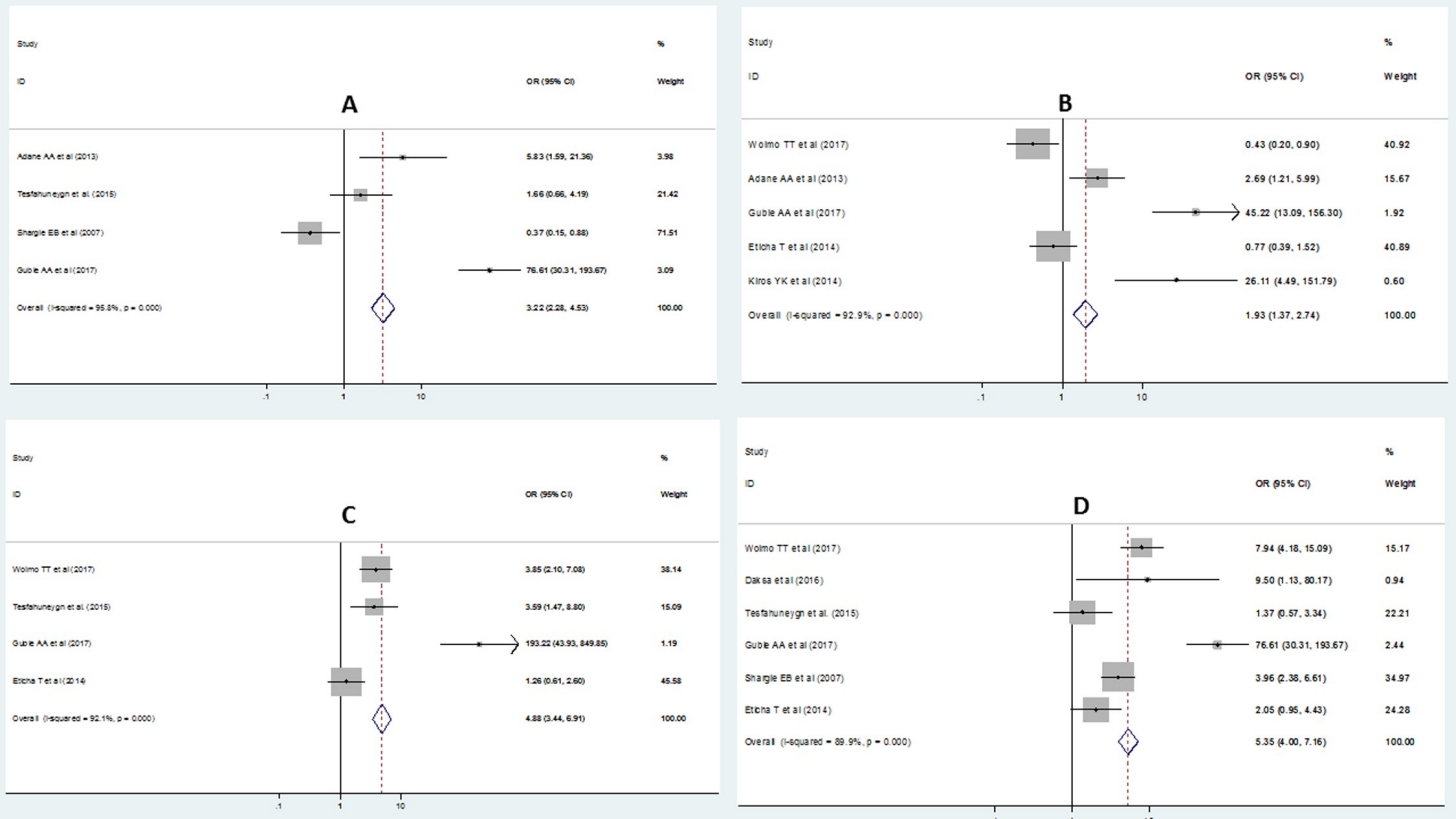
Forest plot depicting pooled odds ratio (log scale) of the associations between non-adherence to Anti TB medication and its purported determinants (A: forgetfulness B: fear of side effect of drugs C: long waiting time in health institution D: feeling distance to health institution is long).

This review assessed the association between forgetfulness of the patients with non-adherence to anti TB medication. The pooled regression analysis of four studies [22, 23, 27, 28] showed a higher risk of non-adherence to TB medication among those who forget to take their medication, OR = 3.22 (95% CI = 2.28, 4.53). The studies included in this analysis were heterogeneous as evidenced by I^2^ (I^2^ = 95.8%, p<0.001) and non-significant publication bias (p-value = 0.790) (Fig 5A).

### Fear of side effect of the drug and non-adherence of anti TB medication

In this subcategory analysis, five articles were included [23, 25, 28–30]. Those studies showed a statistically significant association of fear of side effect of drug with non-adherence of anti TB medication, having higher risk of non-adherence among those who fear side effect of the drugs OR = 1.93 (95% CI = 1.37, 2.74). The studies included in this analysis were heterogeneous as evidenced by I^2^ (I^2^ = 92.9%, p<0.001) and there was no publication bias as indicated by the Egger’s regression test (p-value = 0.076). Therefore, a random effects model was used to estimate the effect of fear of side effect of the drug and non-adherence of anti TB medication (Fig 5B).

### Association between long waiting time (≥1 hour) in health institutions and non-adherence to anti TB treatment

Four studies were included in this category of meta-analysis [22, 23, 25, 30]. The final pooled Meta regression analysis showed statistically significant difference between long waiting time (≥1 hour) in health institutions and non-adherence to anti TB treatment, OR = 4.88 (95% CI = 3.44, 6.91). High heterogeneity was observed in this category of meta-analysis, I^2^ = 92.1%, p<0.001. However, the test for publication bias using the Egger’s test showed non-statistical significant publication bias, p-value = 0.223 (Fig 5C).

### Association between feeling distance to health institution is long and non-adherence to anti TB treatment

In this subcategory analysis, six articles were included [22, 23, 27, 30, 33]. The pooled meta regression analysis showed that there was statistically significant difference in the occurrence of non-adherence to anti TB treatment among those who feel distance to health institution is long, OR = 5.35 (95% CI = 4.00, 7.16)). The studies included in this analysis were heterogeneous as evidenced by (I^2^ = 89.9%, p<0.001) and there was no publication bias as indicated by the Egger’s regression test (p-value = 0.699). Therefore, a random effects model was used to estimate the association between feeling distance to health institution is long and non-adherence to Anti TB treatment (Fig 5D)

## Discussion

Data containing 2797 Ethiopian TB patients were included in the present study. A total of 13 studies were included in the meta-analysis. The study assessed the prevalence of anti TB drug non-adherence and its determinants among TB patients attending Ethiopian health institutions.

Frequently used definition of non-adherence is a patient who had missed 10% or more of the total prescribed doses of TB drugs, even if it is recommended that every TB patient should adhere to anti-TB medication by following the DOTS strategy [1]. Non-adherence to anti-TB treatment might lead to an increased risk of drug resistance and a prolonged infectiousness, in addition to relapse and death. Thus, data obtained from this review is essential for planning, implementing and evaluating advocacy, communication and social mobilization work [39].

The finding from this study showed that 21.29% of TB patients did not adhere to anti TB drugs. Regional variation in non-adherence to anti TB medication was observed, the highest prevalence of non-adherence (18.25%) was reported in Southern Nations and Nationalities of Ethiopia, 23.61% followed by Tigray region with a prevalence of 22.87% and the least was in Amhara studies 10.0%. This can be plausibly explained by the fact that though life in Amhara farming society is hard, many Amhara’s took medication appropriately since they do not want to look ill in front of their families and friends for their better survival. Moreover, resource-limited settings present unique challenges to Anti TB medication adherence. A multitude of structural barriers prevent access to health care and the regular supply of anti-TB drugs. These include the cost of medical care, drugs, and the difficulty of making follow-up appointments due to long distances, family responsibilities and the prohibitive cost of transportation across regions.

This finding is lower compared to the finding from studies conducted in Tertiary health institution in South East Nigeria, in Sub-Saharan Africa and in South Ethiopia where non-adherence to anti TB drug was reported to be 24.2%, 40% and 24.5% respectively [24, 25, 40]. However, these rates are higher than the WHO recommendation of less than 10% [35]. This dissimilarity might be due to socio-cultural differences, variability in TB patient risk factor distribution, and the difference in health service accessibility.

Random effects model was used in our systematic review and meta-analysis, bearing in mind the chances of substantial heterogeneity between studies which were confirmed with the Q test. The pooled prevalence of subgroup analysis revealed clear differences on the prevalence of non-adherence among the regions: Southern Nations and Nationalities of Ethiopia studies reported high prevalence of non-adherence to anti TB treatment followed by Tigray region studies. This discrepancy might be due to the methodological, socio demographic and economic differences between the regions.

It is important to remind that participants were evaluated through self-reported records that measured non-adherence to TB treatment, rather than gold-standard diagnostic clinical interviews in inferring the results of this systematic review and meta-analysis. In addition, even if each self-report measures of non-adherence to TB treatment have limitations, there is indication that among TB patients the lack of anonymity in formal screening assessments may compromise precise assessment of sensitive personal information. Resource-limited settings present unique challenges to anti-TB adherence [18, 19, 21, 33, 41].

Different physical barriers prevent access to health care and the regular supply of anti TB drugs.

Our study have shown that among the Ethiopian TB patients, forgetfulness, fear of side effect of the drug, long waiting time in health institutions and feeling distance to health institution is long were the most significant factors predicting non-adherence which is consistent with earlier studies [42–45].

Our systematic review identified various risk factors associated with non-adherence to anti TB treatment. Most of the factors associated with treatment non-adherence are those related to physical access to health-care services: distance from home to treatment center, employment status, rural residence and a need to use public transport for ambulatory care. In other settings, risk factors such as knowledge about anti TB treatment duration, educational status, poor patient–health provider communication, and medication side effects were reported to have been associated with anti TB treatment non-adherence. This is may be due to less understanding about the disease relapse, resistance and treatment failure in less educated patients. Currently, in Ethiopia, TB treatment is being provided freely at the community, grass root level. However, some patients still questioned the accessibility of TB related services.

Similarly, study conducted in India and Namibia reported that the non-adherence to TB treatment was significantly higher among those who were less educated, unskilled worker, low family income and lower-class family. This may be due to less understanding about the disease relapse, resistance and treatment failure in less educated patients [8, 46]. In this study, forgetfulness showed a significant association with non-adherence to TB treatment. An increment in the overall patient forgetfulness has a positive effect on patient non-adherence to TB treatment. This is consistent with the previous studies in different regions of Ethiopia [26, 41, 47]. This problem may be related to older age which is related with encephalopathy or dementia. From this study long waiting time in health institutions and feeling distance to health institution is long were the main reasons for non-adherence as in line with study done in Peru and Russia in which distance was the main reason for non-adherence followed by feeling better and lack of family support [48, 49]. TB treatment is provided as per DOT principle in Ethiopia, which is difficult for patients to collect their medication every day from the clinics [7]. This suggests that distance from the clinic might be the main barrier for adherence in TB patients. as reported from a study in Southern Ethiopia, side effects of the drugs is also an important reason for anti TB drug non-adherence among TB patients [25]. Whilst the likelihood of not adhering to anti TB drugs among TB patients with side effects of the drugs is almost twice to those without the side effects. This finding is in line with previous reports of studies carried out in Tigray, Northern Ethiopia, where side effects of TB drugs was one of the main reasons for non-adherence among TB patients [30, 32].

### Limitations of the study

The main limitation of this study was that the data were derived from studies that used different screening methods, the prevalence of non-adherence to anti TB medication could not be precisely determined. Second, all of the studies included in this review were cross-sectional in design; it neither represents seasonal variation of non-adherence outcomes nor establishes causal relationship. In addition, only articles published in English language were considered to include in this review. It should also be noted that some of the studies included in this review had a small sample size which could affect the estimated report.

## Conclusion

In this meta-analysis, studies revealed that the prevalence of non-adherence to anti-TB drugs among TB patients in Ethiopia was relatively high. Forgetfulness, fear of side effect of the drug, long waiting time in health institutions and feeling distance to health institution is long were significant determinants of non-adherence to anti-TB drugs. Therefore, based on our findings, we recommend that policies should be developed to strengthen the treatment management of TB patients. Where possible, decentralization of DOTs service to lower level of health facility structure should be prioritized.Moreover, side effects and other reasons which account for missing medication should be monitored early so that patients will adhere more to their medications.

## Supporting information

S1 File supplementary file search strategy. (DOCX)

## Abbreviations

DOT: Directly Observed Treatment
TB: Tuberculosis
HIV: Human Immunodeficiency Virus
MDR-TB: Multi Drug Resistance–Tuberculosis
WHO: World Health Organization

## References

[1] Organization WH. Global tuberculosis report 2016. 2016.

[2] Dye C, Scheele S, Dolin P, Pathania V, Raviglione MC. Global burden of tuberculosis: estimated incidence, prevalence, and mortality by country. Jama. 1999;282(7):677–86. 10517722 10.1001/jama.282.7.677

[3] Floyd K, Glaziou P, Zumla A, Raviglione M. The global tuberculosis epidemic and progress in care, prevention, and research: an overview in year 3 of the End TB era. The Lancet Respiratory Medicine. 2018;6(4):299–314. 10.1016/S2213-2600(18)30057-2 29595511

[4] Ahlburg DA, Initiative ST. The economic impacts of tuberculosis. 2000.

[5] Organization WH. Global tuberculosis control: epidemiology, strategy, financing: WHO report 2009: World Health Organization; 2009.

[6] Organization WH. Global tuberculosis report 2013: World Health Organization; 2013.

[7] FMoH E. Tuberculosis, Leprosy and TB/HIV Prevention and Control Programme. Addis Ababa, Ethiopia 2008;207.

[8] Chani K. Factors affecting compliance to tuberculosis treatment in Andara Kavango region Namibia 2010.

[9] Rieder HL. Interventions for tuberculosis control and elimination: International Union Against Tuberculosis and Lung Disease Paris; 2002.

[10] Sabaté E. Adherence to long-term therapies: evidence for action: World Health Organization; 2003. 14562485

[11] Harries AD, Maher D, Graham S. TB/HIV: a clinical manual: World Health Organization; 2004.

[12] Awofeso N. Anti-tuberculosis medication side-effects constitute major factor for poor adherence to tuberculosis treatment. Bulletin of the World Health Organization. 2008;86:B-D. 10.2471/BLT.07.043802 PMC2647396 18368191

[13] Organization WH. WHO report 2008: global tuberculosis control: surveillance, planning, financing. WHO report 2008: global tuberculosis control: surveillance, planning, financing2008.

[14] Obermeyer Z, Abbott-Klafter J, Murray CJ. Has the DOTS strategy improved case finding or treatment success? An empirical assessment. PLoS One. 2008;3(3):e1721 10.1371/journal.pone.0001721 18320042 PMC2253827

[15] Yin X, Tu X, Tong Y, Yang R, Wang Y, Cao S, et al Development and validation of a tuberculosis medication adherence scale. PLoS One. 2012;7(12):e50328 10.1371/journal.pone.0050328 23251363 PMC3520953

[16] Habteyes Hailu T, Azar T, Davoud SHOJAEIZADEH GG. Tuberculosis treatment non-adherence and lost to follow up among TB patients with or without HIV in developing countries: a systematic review. Iranian journal of public health. 2015;44(1):1 26060770 PMC4449995

[17] Fox W. Compliance of patients and physicians: experience and lessons from tuberculosis-II. British medical journal (Clinical research ed). 1983;287(6385):101. 6407697 10.1136/bmj.287.6385.101 PMC1548343

[18] Gebremariam MK, Bjune GA, Frich JC. Barriers and facilitators of adherence to TB treatment in patients on concomitant TB and HIV treatment: a qualitative study. BMC public health. 2010;10(1):651. 21029405 10.1186/1471-2458-10-651 PMC2978153

[19] Gele AA, Sagbakken M, Abebe F, Bjune GA. Barriers to tuberculosis care: a qualitative study among Somali pastoralists in Ethiopia. BMC research notes. 2010;3(1):86. 20353599 10.1186/1756-0500-3-86 PMC2853549

[20] Sagbakken M, Frich JC, Bjune G. Barriers and enablers in the management of tuberculosis treatment in Addis Ababa, Ethiopia: a qualitative study. BMC public health. 2008;8(1):11. 18186946 10.1186/1471-2458-8-11 PMC2257959

[21] Bagchi S, Ambe G, Sathiakumar N. Determinants of poor adherence to anti-tuberculosis treatment in Mumbai, India. International journal of preventive medicine. 2010;1(4):223 21566777 PMC3075517

[22] Tesfahuneygn G, Medhin G, Legesse M. Adherence to Anti-tuberculosis treatment and treatment outcomes among tuberculosis patients in Alamata District, northeast Ethiopia. BMC research notes. 2015;8(1):503. 26420164 10.1186/s13104-015-1452-x PMC4588463

[23] GUBE AA, DEBALKIE M, SEID K, BISETE K, MENGESHA A, ZEYNU A, et al. ASSESSMENT OF ANTI TB DRUG NON-ADHERENCE AND ITS ASSOCIATED FACTORS AMONG TB PATIENTS ATTENDING TB CLINICS IN ARBA MINCH GOVERNMENTAL HEALTH INSTITUTIONS, SOUTHERN ETHIOPIA. 10.1155/2018/3705812 PMC5835254 29670768

[24] Yusuf KO, Seifu MF, Gelaw BK, Gebremariam ET. Non Adherence and its Contributing Factors to Anti-TB Drug in Childrenâ€ s at Adama Referral Hospital, Oromia, Ethiopia. Global Journal of Medical Research. 2015.

[25] Woimo TT, Yimer WK, Bati T, Gesesew HA. The prevalence and factors associated for anti-tuberculosis treatment non-adherence among pulmonary tuberculosis patients in public health care facilities in South Ethiopia: a cross-sectional study. BMC public health. 2017;17(1):269 10.1186/s12889-017-4188-9 28320351 PMC5359861

[26] Kebede A, Wabe NT. Medication adherence and its determinants among patients on concomitant tuberculosis and antiretroviral therapy in South West Ethiopia. North American journal of medical sciences. 2012;4(2):67 10.4103/1947-2714.93376 22408750 PMC3296321

[27] Shargie EB, Lindtjørn B. Determinants of treatment adherence among smear-positive pulmonary tuberculosis patients in Southern Ethiopia. PLoS medicine. 2007;4(2):e37 10.1371/journal.pmed.0040037 17298164 PMC1796905

[28] Adane AA, Alene KA, Koye DN, Zeleke BM. Non-adherence to anti-tuberculosis treatment and determinant factors among patients with tuberculosis in northwest Ethiopia. PloS one. 2013;8(11):e78791 10.1371/journal.pone.0078791 24244364 PMC3823971

[29] Kiros Y, Teklu T, Desalegn F, Tesfay M, Klinkenberg E, Mulugeta A. Adherence to anti-tuberculosis treatment in Tigray, Northern Ethiopia. Public health action. 2014;4(3):S31–S6. 26478511 10.5588/pha.14.0054 PMC4542072

[30] Eticha T, Kassa E. Non-Adherence to Anti-TB Drugs and Its Predictors among TB/HIV Co-Infected Patients in Mekelle, Ethiopia. Journal of Bioanalysis and Biomedicine. 2014;6(6):61.

[31] Nezenega ZS, Tafere TE. Patient satisfaction on tuberculosis treatment service and adherence to treatment in public health facilities of Sidama zone, South Ethiopia. BMC health services research. 2013;13(1):110. 23521921 10.1186/1472-6963-13-110 PMC3658999

[32] al. Ge. LEVEL OF PATIENT ADHERENCE TO ANTI-TUBERCULOSIS TREATMENT IN MEKELLE TUBERCULOSIS DIRECT OBSERVED THERAPY CENTERS, TIGRAY, NORTH ETHIOPIA. WORLD JOURNAL OF PHARMACY AND PHARMACEUTICAL SCIENCES. 2015;4(04):166–81.

[33] al De. Patients’ adherence to anti-tuberculosis medicines and associated factors for non-adherence at a tertiary teaching hospital, South West Ethiopia. Gaziantep Medical Journal 2016;22(2):55–62.

[34] al AYe. Treatment Adherence among Tuberculosis and Human Immuno Deficiency Virus Coinfected Patients in Ginnir Referral Hospital. American Journal of Public Health Research. 2014;2(6):239–43.

[35] Organization WH. World Health Organization global tuberculosis control report 2009. Global tuberculosis control. 2011.

[36] Liberati A, Altman DG, Tetzlaff J, Mulrow C, Gøtzsche PC, Ioannidis JP, et al The PRISMA statement for reporting systematic reviews and meta-analyses of studies that evaluate health care interventions: explanation and elaboration. PLoS medicine. 2009;6(7):e1000100 10.1371/journal.pmed.1000100 19621070 PMC2707010

[37] Higgins JP, Thompson SG, Deeks JJ, Altman DG. Measuring inconsistency in meta-analyses. BMJ: British Medical Journal. 2003;327(7414):557 10.1136/bmj.327.7414.557 12958120 PMC192859

[38] Shargie EB, Lindtjorn B. Determinants of treatment adherence among smear-positive pulmonary tuberculosis patients in Southern Ethiopia. PLoS Med. 2007;4(2):e37 10.1371/journal.pmed.0040037 17298164 PMC1796905

[39] Haynes RB, McDonald H, Garg AX, Montague P. Interventions for helping patients to follow prescriptions for medications. Cochrane Database Syst Rev. 2002;2(11):7.9–1.4. 10.1002/14651858.CD000011 12076376

[40] Ubajaka CF, Azuike EC, Ugoji JO, Nwibo OE, Ejiofor OC, Modebe IA, et al Adherence to Drug Medications amongst Tuberculosis Patients in a Tertiary Health Institution in South East Nigeria. International Journal of Clinical Medicine. 2015;6(06):399.

[41] Abula T, Worki A. Patient non-compliance with drug regiments for chronic diseases in northwest Ethiopia. Ethiopian Journal of Health Development. 2001;15(3):185–92.

[42] Michael K, Belachew T, Jira C. Tuberculosis defaulters from the" dots" regimen in Jimma zone, southwest Ethiopia. Ethiopian medical journal. 2004;42(4):247–53. 16124124

[43] Tekle B, Mariam D, Ali A. Defaulting from DOTS and its determinants in three districts of Arsi Zone in Ethiopia. The International Journal of Tuberculosis and Lung Disease. 2002;6(7):573–9. 12102295

[44] Munro SA, Lewin SA, Smith HJ, Engel ME, Fretheim A, Volmink J. Patient adherence to tuberculosis treatment: a systematic review of qualitative research. PLoS medicine. 2007;4(7):e238 10.1371/journal.pmed.0040238 17676945 PMC1925126

[45] Frieden TR, Sbarbaro JA. Promoting adherence to treatment for tuberculosis: the importance of direct observation. Bulletin of the World Health Organization. 2007;85:407–9. 10.2471/06.038927 17639230 PMC2636637

[46] Santha T, Garg R, Frieden T, Chandrasekaran V, Subramani R, Gopi P, et al Risk factors associated with default, failure and death among tuberculosis patients treated in a DOTS programme in Tiruvallur District, South India, 2000. The International Journal of Tuberculosis and Lung Disease. 2002;6(9):780–8. 12234133

[47] Adane K, Ameni G, Bekele S, Abebe M, Aseffa A. Prevalence and drug resistance profile of Mycobacterium tuberculosis isolated from pulmonary tuberculosis patients attending two public hospitals in East Gojjam zone, northwest Ethiopia. BMC public health. 2015;15:572 10.1186/s12889-015-1933-9 26092570 PMC4473837

[48] Culqui DR, Grijalva CG, Cayla JA, Horna-Campos O, Ch KA. Factors associated with the non-completion of conventional anti-tuberculosis treatment in Peru. Archivos de Bronconeumología (English Edition). 2012;48(5):150–5. 10.1016/j.arbres.2011.12.008 22377140

[49] Gelmanova I, Keshavjee S, Golubchikova V, Berezina V, Strelis AK, Yanova GV, et al Barriers to successful tuberculosis treatment in Tomsk, Russian Federation: non-adherence, default and the acquisition of multidrug resistance. Bulletin of the World Health Organization. 2007;85(9):703–11. 10.2471/BLT.06.038331 18026627 PMC2636414

