# Supplementary material for: Prevalence and determinants of anti-tuberculosis treatment non-adherence in Ethiopia: A systematic review and meta-analysis
Source: PLoS One. 2019 Jan 10;14(1):e0210422. doi: 10.1371/journal.pone.0210422 (PMC6328265; doi:10.1371/journal.pone.0210422)
Supplement: S1 File — (DOCX) [file pone.0210422.s001.docx]

***Search protocol***

**Supplementary material.** Data sources and search criteria for systematically reviewing literature reporting on Tuberculosis treatment Non adherence and associated factors in Ethiopia.

**Medline/PubMed**

**The final search protocol for Medline from 1980 to February, 2018 limited to studies on human subjects**

***Literature search string using variant Mesh and Text terms* *combined***

The search strategy that had been used to identify studies of the Tuberculosis treatment Non adherence and its determinant factors:

1. TB AND Non-adherence OR adherence.
2. TB AND risk factor(s).
3. TB AND Ethiopia.

((‘Tuberculosis’ [MeSH Terms]) OR ‘TB’) AND ‘adherence to TB medication’ [MeSH Terms]) OR ‘non-adherence TB medication’ [MeSH Terms]) AND ‘Ethiopia’) AND ‘associated factors’) OR ‘determinants’ AND prevalence) OR epidemiology)

**Cochrane library**

1. MeSH descriptor: [Tuberculosis] explode all trees, 2.TB*: ti,ab,kw (Word variations have been searched), 3.MeSH descriptor: [Non adherence] explode all trees, 4.adherance:ti,ab,kw (Word variations have been searched), 5.MeSH descriptor: [associated factors] explode all trees, 6.MeSH descriptor: [Prevalence] explode all trees, 7.MeSH descriptor: [Epidemiology] explode all trees, 8.MeSH descriptor: [Ethiopia] explode all trees, 9. ((#1 or #2) and (#3 or #4) and #5 and (#6 or #7) and #8)

**Embase**

((TB:ab,ti OR ‘TB infection’:ab,ti OR ‘Tuberculosis’:ab,ti ‘TB’/exp OR ‘TB infection’/exp OR ‘Tuberculosis’/exp )AND (‘non adherence’:ab,ti OR ‘adherence:ab,ti OR ‘medication adherence’:ab,ti OR ‘medication non adherence’:ab,ti non adherence’/exp OR ‘adherence/exp OR ‘medication adherence’/exp OR ‘medication non adherence’/exp) AND (‘Ethiopia’:ab,ti ‘Ethiopia’/exp) AND (‘associated factors’ ab,ti OR ‘determinants’ab,ti OR ‘predictors’ ab,ti ‘associated factors’/exp OR ‘determinants’/exp OR ‘predictors’/exp) AND (‘Prevalence’ ab,ti ‘Prevalence’/exp) OR ‘Epidiomology‘ab,ti ‘Epidiomology’/exp))
